# Motor Imagery EEG Signal Classification Using Distinctive Feature Fusion with Adaptive Structural LASSO

**DOI:** 10.3390/s24123755

**Published:** 2024-06-09

**Authors:** Weihai Huang, Xinyue Liu, Weize Yang, Yihua Li, Qiyan Sun, Xiangzeng Kong

**Affiliations:** 1 College of Mechanical and Electrical Engineering, Fujian Agriculture and Forestry University, Fuzhou 350100, China; 1221298003@fafu.edu.cn (W.H.); 52312047021@fafu.edu.cn (W.Y.); 52312048024@fafu.edu.cn (Y.L.); 2 School of Future Technology, Fujian Agriculture and Forestry University, Fuzhou 350002, China; 5226243003@fafu.edu.cn; 3 College of Computer and Information Sciences, Fujian Agriculture and Forestry University, Fuzhou 350002, China

**Keywords:** electroencephalography, brain–computer interface, motor imagery, common spatial pattern, adaptive LASSO

## Abstract

A motor imagery brain–computer interface connects the human brain and computers via electroencephalography (EEG). However, individual differences in the frequency ranges of brain activity during motor imagery tasks pose a challenge, limiting the manual feature extraction for motor imagery classification. To extract features that match specific subjects, we proposed a novel motor imagery classification model using distinctive feature fusion with adaptive structural LASSO. Specifically, we extracted spatial domain features from overlapping and multi-scale sub-bands of EEG signals and mined discriminative features by fusing the task relevance of features with spatial information into the adaptive LASSO-based feature selection. We evaluated the proposed model on public motor imagery EEG datasets, demonstrating that the model has excellent performance. Meanwhile, ablation studies and feature selection visualization of the proposed model further verified the great potential of EEG analysis.

## 1. Introduction

The brain–computer interface (BCI) is a system that converts brain neural signals into external device control instructions, enabling people to control surrounding devices or communications [1]. In BCI systems, motor imagery based on electroencephalography (EEG) represents one of the most pivotal patterns. By analyzing the physiological information related to limb movements in EEG signals, the motor imagery BCI realizes brain–computer communication and can be used in auxiliary rehabilitation training [2,3,4], virtual games [5], navigation [6] and other scenarios. Given the broad prospects, the motor imagery BCI has attracted widespread attention around the world.

In recent years, machine learning has been favored in the motor imagery EEG signal classification due to its good ability to mine task-relevant features in high-dimensional data [7,8]. Analysis methods based on machine learning can be divided into three parts: feature extraction, feature selection, and classification. Task-relevant feature extraction is the basis for subsequent steps. However, discriminative features in EEG signals are easily masked by other physiological signals and are difficult to obtain directly [9]. In order to achieve accurate classification, a large number of feature extraction methods are used to extract relevant features. Among them, feature extraction methods based on deep learning are a type of end-to-end method driven by data. Through the combination of neural network layers with different parameters, deep learning methods automatically mine nonlinear features with task-relevant information in EEG signals and identify motor imagery tasks [10,11,12,13]. However, deep learning methods rely on training with large amounts of samples and are susceptible to data perturbations [14].

Traditional machine learning-based methods usually introduce prior knowledge to extract task-related features. The physiological information contained in EEG is sparsely distributed in high-dimensional signal data, which is not conducive to classification by classifiers. Therefore, analysis methods, such as time domain analysis methods, frequency domain analysis methods, and time-frequency domain analysis methods, are used to extract the features of EEG signals, re-characterizing the task-related information. In time domain analysis, data are usually reduced to lower dimensions by extracting features, such as statistical features and the Hjorth parameters [15,16]. In frequency domain analysis, analysis methods, such as fast Fourier transform and power spectral density, re-represent EEG signals by extracting frequency domain features [17,18]. In order to improve the resolution of EEG signals, time–frequency analysis methods, such as wavelet transform [19], empirical mode decomposition [20], complex variational mode decomposition [21] and Hilbert–Huang transform [22,23], are used to decompose the original signal into different sub-bands and extract features from sub-band signals. Yu et al. [21] decomposed EEG signals into band-limited sub-signals and extracted features of the sub-signals. However, these three types of methods are difficult to flexibly handle nonlinear signals such as EEG signals and are often limited by prior knowledge and manual parameters.

Multi-channel EEG signals are collected from different encephalic regions and contain spatial information about brain activity changes in different regions during motor imagery. Extracting features from the spatial domain has been shown to be effective in EEG signal classification [24,25]. Common spatial pattern (CSP) is the most commonly used spatial domain feature extraction method and is considered to be one of the most effective feature extraction methods in motor imagery classification [24]. By finding a set of spatial filters that maximize the covariance difference between different classes, CSP successfully extracts spatial domain features with distinctive information from multi-channel EEG signals [9,26]. Other spatial domain feature extraction methods such as hierarchical discriminant component analysis are also widely used in motor imagery [27].

Usually, spatial domain analysis methods are applied to EEG signals with a wide frequency range or manually selected specific frequency ranges, making it difficult to flexibly learn subject-specific discriminative features. Considering the individual differences in the frequency ranges of brain activity, Ang et al. [25] used the CSP algorithm to obtain the spatial features of sub-band signals with non-overlapping frequency bands and performed feature selection on the extracted features. Although signal decomposition enables the CSP algorithm to achieve better discriminative feature extraction, this band segmentation strategy is still insufficient for the model to match the frequency range of a specific subject’s brain activity during motor imagery. To match the features to the specific subject, we introduced a joint frequency-domain and spatial-domain feature extraction strategy for the CSP algorithm that decomposed EEG signals into multi-scale and overlapping sub-bands and expected to use feature selection methods to fuse the multi-scale spatial features.

Feature selection is used to identify and select the features that contribute the most to the task from the generated features, optimizing model performance and reducing computational complexity. Generally, feature selection methods can be divided into filter methods, wrapper methods and embedded methods. Filter methods are a type of method that implements feature sorting and screening by analyzing the data itself [28]. It is independent of the learning algorithm and has a low computational cost. Wrapper methods train classifiers with different feature subsets and evaluate the subsets through classifier performance metrics [29]. Compared to filter methods and wrapper methods, embedded methods offer a distinct approach by integrating the feature selection process into the training of machine learning models. In embedded methods, the algorithms perform feature selection while training the model, which can more effectively capture the dependencies and interactions between features.

The feature selection methods based on the least absolute shrinkage and selection operator (LASSO) algorithm are excellent embedded methods that aim to filter out irrelevant and redundant features [30]. Their feature selection process is embedded into the classifier training process, achieving excellent feature selection performance while reducing computational costs. Miao et al. [31] introduced the LASSO-based feature selection into the motor imagery model for selecting salient features and achieved excellent performance. To automatically select the subjects with the greatest contribution for further sparse representation, Jiao et al. [32] introduced the sparse group LASSO-based feature selection into the motor imagery classification, achieving both intra-group and inter-group sparsity. The feature selection based on the LASSO algorithms has achieved good performance on motor imagery classification. However, the LASSO-based feature selection in motor imagery classification models only considers sparsity and ignores the exclusion of some discriminative features. To select discriminative features reliably and robustly, we introduced the symmetric uncertainty and the spatial information of features into LASSO. We expected to improve the selective ability of the LASSO-based feature selection by accurately evaluating feature importance and adaptively mining structural information between features.

In this paper, we proposed a flexible motor imagery EEG signal classification model using distinctive feature fusion with adaptive structural LASSO (AS-LASSO) to extract features matching specific subjects. We extracted the spatial domain features from overlapping and multi-scale sub-bands of EEG signals through the CSP algorithm. Then, the AS-LASSO-based feature selection was performed to select the optimal feature subset. AS-LASSO introduced the symmetric uncertainty and the spatial information of features to evaluate the penalty weight of adaptive LASSO, which enables the model to more accurately select discriminative features and fully consider the complementarity between features. Finally, the optimal subset was fed to the support vector machine (SVM) to implement motor imagery classification. The proposed model was validated on public EEG datasets, demonstrating that the model outperformed state-of-the-art models in motor imagery classification. Moreover, we performed ablation experiments and visualized selected features, further validating the potential of the proposed model.

The main contributions of this paper are summarized as follows:

1. A flexible feature learning model for motor imagery EEG signal classification, namely frequency-spatial feature fusion (FSFF), is proposed. By using distinctive feature fusion with AS-LASSO, the model can flexibly capture multi-scale spatial information matched to a specific subject.
2. A joint frequency-domain and spatial-domain feature extraction strategy is developed for the CSP algorithm. By setting up a set of overlapping bandpass filters, we extracted spatial domain features at multiple scales to match the specific subject.
3. A novel feature selection algorithm is constructed that introduces the symmetric uncertainty and spatial information of features into adaptive LASSO, namely AS-LASSO. It can accurately select discriminative features and fully utilize the complementarity between features by mining task relevance and structural information.
4. Experiments on multiple public EEG datasets demonstrate that the proposed model excels in efficiently extracting discriminative features, potentially enhancing the flexibility and accuracy of EEG analysis. At the same time, it proves that the model provides a robust tool for BCI applications, such as auxiliary rehabilitation training.

The rest of this paper is organized as follows. We presented the related work in Section 2 and introduced the details of our proposed model in Section 3. In Section 4, we introduced the experimental details and discussed the experimental results of our model. Finally, some conclusions were presented in Section 5.

## 2. Related Work

In this section, we reviewed recent advances in discriminative feature extraction and LASSO-based feature selection.

### 2.1. Discriminative Feature Extraction

In traditional EEG analysis algorithms, time domain methods [16], frequency domain methods [17,18], time–frequency domain methods [19,20,22,23] and spatial domain methods [9,24,26,27] are usually used to extract task-relevant features of EEG signals. These manual feature extraction methods are often limited by prior knowledge. Khorshidtalab et al. [15] improved the two time-domain features of Wilson amplitude and slope sign change to overcome the shortcomings of determining appropriate thresholds through repeated trials and achieved good motor imagery classification performance. To extract local information from EEG signals, Wagh et al. [33] decomposed the signal into multiple frequency bands through discrete wavelet transform to derive various features such as energy, standard deviation, and variance. Improving resolution and overcoming manual parameter limitations have attracted much attention as a means to improve feature quality. However, the significant frequency bands of brain activity differ between individuals during motor imagery tasks, making it difficult to match the features extracted in the time domain and time-frequency domain to a specific subject.

Spatial domain methods typically analyze differences in brain activity of different brain areas during performing motor imagery tasks [24]. Among them, CSP is a commonly used spatial feature extraction method for individuals. It mainly processes the multi-channel spatial distribution of EEG signals by designing spatial filters that maximize the signal variance of the two-category classification task [24]. To avoid inaccuracies and the loss of important information, Barachant et al. [34] introduced Riemannian geometry to calculate the variance in the CSP algorithm. However, the Riemannian geometry-based methods cause the model calculation cost to increase dramatically in high dimensions [35].

Taking into account the individual differences in the frequency ranges of brain activity, Ang et al. [25] proposed an optimized CSP algorithm to implement an autonomous selection of discriminative spatial features, which is called filter bank common spatial pattern (FBCSP). By decomposing EEG signals into non-overlapping sub-bands of different frequency ranges, FBCSP improved the resolution of EEG spatial information and the adaptive learning ability of CSP. However, this rough decomposition method destroys the important information in EEG signals and is difficult to adapt for specific subjects. To enable the model to flexibly extract EEG spatial features that match the subject, we decomposed EEG signals into sub-bands of different frequency scales and different frequency ranges with overlapping frequency bands and fused multi-scale spatial features through feature selection.

### 2.2. LASSO-Based Feature Selection

Processing high-dimensional features often leads to increased computational complexity and results in the curse of dimensionality, which reduces model efficiency and increases the challenge of building effective classifiers. Therefore, feature selection is widely used in learning models. It is crucial to reduce data dimensionality and the risk of overfitting.

Commonly, feature selection methods can be divided into filter methods, wrapper methods, and embedded methods. Filter methods evaluate the importance of features through evaluation criteria such as mutual information and *t*-tests and select features based on their scores [36]. Filter methods have the characteristic of low computational cost. However, they only consider the impact of single features, ignoring the interaction between features. Wrapper methods provide a class of methods that rely on the performance metrics of classifiers trained on different feature subsets to select the optimal subset. Although wrapper methods outperform filter methods in terms of performance, they often require higher computational costs due to their repeated construction of classifiers [29]. Embedded methods integrate the feature selection process into the model training, providing efficient methods for identifying discriminative features. Compared with the first two types of methods, embedded methods not only consider the correlation between features but also avoid the large computational cost caused by repeatedly training classifiers.

LASSO is an embedded method for simultaneous estimation and selection, which has the advantage of balancing feature selection performance and computational cost [30]. However, LASSO does not have oracle properties. It tends to arbitrarily select a few variables from a group of highly correlated ones, resulting in low robustness. To avoid important information being excluded, Yuan and Lin [37] proposed an optimized version of LASSO that introduced prior grouping into the LASSO algorithm, which is called group-LASSO. Subsequently, Simon et al. [38] further proposed sparse group-LASSO, which not only achieved inter-group sparsity but also considered intra-group sparsity. The group-based LASSO algorithms have been applied to EEG feature selection and have achieved good performance improvements [39]. However, the group-based LASSO algorithms rely on group division. When groupings are inconsistent, the group-based LASSO algorithms may not be fully applicable. Moreover, the estimates obtained by using the same adjustment parameter for penalizing all coefficients may also be significantly biased.

Zou [40] deduced the conditions for LASSO to have oracle properties and proposed another optimization idea for the LASSO algorithm, which is called adaptive LASSO. By weighting the penalty terms to different degrees, the variable coefficients in the LASSO algorithm have different degrees of penalty, achieving faster and more stable estimates. Based on the idea of adaptive penalty, a variety of penalty weight measurement strategies, such as methods based on correlation, rank, and *t*-test, have been proposed [41,42]. Strategies, such as using the Pearson correlation coefficient, make feature selection more targeted and avoid the bias introduced by the LASSO-based weight measurement that exists in the original adaptive LASSO. However, these strategies are easily affected by noise and fail to further consider feature-related prior knowledge.

Considering the characteristics of low amplitude and low signal-to-noise ratio of EEG signals, we chose the symmetric uncertainty to optimize adaptive LASSO. Symmetric uncertainty is an indicator of modified mutual information, which can robustly reflect the relationship between features and class labels [43]. Furthermore, we introduced the spatial information of features into adaptive LASSO to provide structural information. In addition to the task relevance of features, external structural information also contributes to feature selection. Prior structural information enables the model to consider multiple sets of structures that exist between features and avoids bias caused by introducing group structure. By combining the symmetric uncertainty and the spatial information of features, AS-LASSO can fully utilize the task relevance and structure information of features.

In this work, we extracted the multi-scale spatial features of EEG signals through the CSP algorithm and mined discriminative features using the AS-LASSO-based feature selection. By fusing spatial features of different ranges and different scales, the proposed model was able to fully utilize the complementarity of multi-scale features and capture discriminative features matched to the specific subject.

## 3. Methods

**Figure 1:**
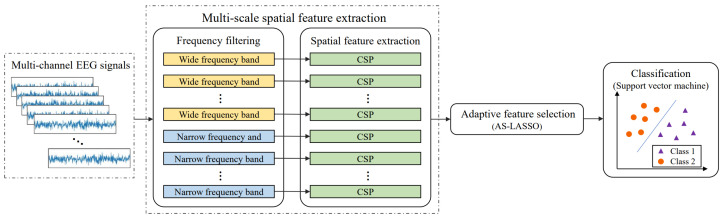
Flowchart of the proposed model.

In this section, we introduced our proposed model, as shown in Figure 1. First, we extracted the multi-scale spatial features of EEG signals through the CSP algorithm. Then, the AS-LASSO-based feature selection was performed to select features with high matching degrees for the specific subject. To adaptively select discriminative features, we introduced the symmetric uncertainty and the spatial information of features to adaptive LASSO, which not only effectively estimates task relevance but also utilizes latent structural information. Finally, the optimal feature subset was fed into the SVM classifier for classification.

### 3.1. Multi-scale Spatial Feature Extraction

As a spatial domain feature extraction method, CSP has been widely used in motor imagery [35]. The CSP algorithm effectively mines the spatial information in EEG signals that can reflect the brain’s intention by setting a set of spatial filters and avoids the influence of differences in EEG signals between individuals. However, the effective application of the CSP algorithm depends on the setting of EEG signal frequency ranges, which makes it difficult to match different subjects.

**Figure 2:**
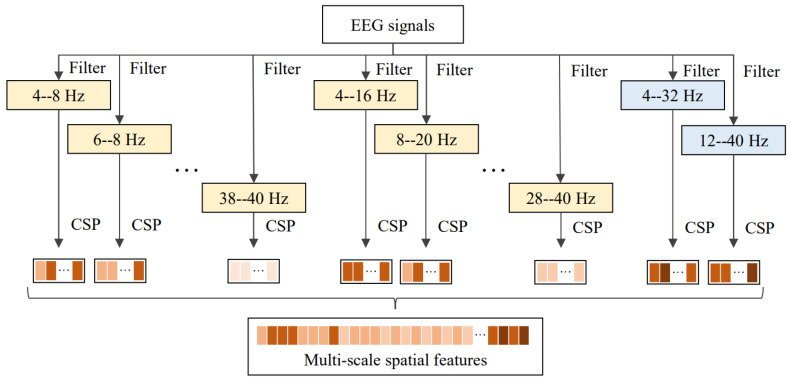
Flowchart of the multi-scale spatial feature extraction.

In this work, we introduced a joint frequency domain and spatial domain feature extraction strategy for the CSP algorithm that decomposed EEG signals into overlapping sub-bands with different frequency scales and different frequency ranges. This decomposition aims to capture spatial features tailored to specific subjects within the motor imagery classification. EEG signals are segmented into overlapping sub-bands of different frequency bands, and their spatial features are then calculated through the CSP algorithm. Based on previous research, we set the frequency bands to be in the range of 4∼40 Hz [25]. Specifically, we had set up two types of frequency bands: wide frequency bands and narrow frequency bands, as shown in Figure 2. The extracted feature set was recorded as $X=\left(x_{1},x_{2},\ldots ,x_{p}\right)\in R^{n\times p}$, where *n* represents the number of samples and *p* represents the number of features. Each $x_{i}={\left(x_{1i},x_{2i},\ldots ,x_{ni}\right)}^{T}\in R^{n\times 1}$ is a vector of a feature. Through the settings of different frequency bands, the model can learn information at different scales at the same time, allowing it to better match the brain activity of a specific subject.

### 3.2. Adaptive Feature Selection

Through multi-scale spatial feature extraction, we captured information at different frequency scales of EEG signals. However, redundant features and features contaminated by noise can easily affect model construction and limit its application. Therefore, we proposed a novel feature selection method based on adaptive LASSO to improve model performance by selecting discriminative features.

#### 3.2.1. Weight Measurement

EEG signals have the characteristic of a low signal-to-noise ratio, making it difficult to extract signal features without noise interference. To robustly measure the weight of penalty terms, we used the symmetric uncertainty and the spatial information of features to evaluate the contributions of the features to the model.

Symmetric uncertainty is an information entropy-based relevance measurement approach that is robust to noise. By correcting for mutual information, symmetric uncertainty avoids the disadvantage of mutual information’s tendency for multi-valued features [43]. We computed the symmetric uncertainty between features and class labels as task relevance of feature. The symmetric uncertainty of the *i*-th feature $x_{i}$ and the label vector $y={\left(y_{1},y_{2},\ldots ,y_{n}\right)}^{T}\in R^{n\times 1}$ is calculated as follows:

(1) $$s_{i}=\frac{2I(x_{i};y)}{H\left(x_{i}\right)+H\left(y\right)}$$

where $H(x_{i})$ is the information entropy of $x_{i}$, H(y) is the information entropy of y, and $I(x_{i};y)$ is the mutual information of $x_{i}$ and y. The symmetric uncertainty normalizes values between 0 and 1. When the value is 0, it means that $x_{i}$ and y are irrelevant. When the value is 1, it indicates that $x_{i}$ and y are perfectly correlated.

In addition, we introduced the spatial information of features to assist the LASSO-based feature selection to mine the structures of features. CSP uses the diagonalization of the matrix to find a set of spatial filters for projection to maximize the difference in the variance of two types of signals. According to previous research, we used the eigenvalues obtained from the decomposition of the whitened covariance matrix when constructing spatial filters as the spatial information of features. Typically, eigenvalues that are related to the significance of brain activity at different channel locations are used to select the best channels or filter banks [44,45]. We calculated the absolute difference from the eigenvalues to the mean, which is recorded as $e=\left(e_{1},e_{2},\ldots ,e_{p}\right)\in R^{p}$. $e_{i}$ is the spatial information of the *i*-th feature, which reflects the significance of its corresponding feature in the spatial domain.

According to the basic assumption of adaptive LASSO, the penalty weight should be inversely proportional to feature importance [40]. We combined the symmetric uncertainty and the spatial information of the feature and adjusted it through an exponential function to obtain the penalty weight. The penalty weight is calculated as follows:

(2) $$w_{i}=e^{-\mu s_{i}e_{i}}$$

where μ is the adjustment parameter. The calculation of the penalty term weight based on the symmetric uncertainty and the spatial information reduces the impact of noise and enables the algorithm to mine potential structural relationships between features.

#### 3.2.2. LASSO-Based Feature Selection

Considering that the LASSO-based feature selection has the advantages of balancing performance and computational cost, we applied the LASSO-based feature selection to the motor imagery classification model. LASSO is a linear regression method for simultaneous estimation and variable selection [30]. By introducing the L1 penalty term, LASSO compresses a part of the coefficients to zero while estimating the variable coefficients. The object function of LASSO can be written as

(3) $$\min_{\beta }{\{∥y-X\beta ∥}_{2}^{2}+{\lambda ∥\beta ∥}_{1}\}$$

where $\beta ={\left(\beta_{1},\beta_{2},\ldots ,\beta_{p}\right)}^{T}\in R^{p}$ is an unknown vector of regression coefficients, and λ is the penalty parameter used to adjust the degree of penalty. When LASSO is applied to feature selection, features with coefficients of 0 are excluded, and features with non-zero coefficients are selected. However, LASSO does not have oracle properties. Feature subsets selected by LASSO are consistent only under certain conditions.

Zou [40] deduced the conditions for LASSO to have oracle properties and proposed an optimized version of the LASSO algorithm, called adaptive LASSO. Its objective function can be written as

(4) $$\min_{\beta }{\{∥y-X\beta ∥}_{2}^{2}+\lambda \sum_{i=1}^{p}w_{i}|\beta_{i}\left|\right\}$$

Suppose that $\hat{\beta }$ is a root-n-consistent estimator to β; for example, we can use $\hat{\beta }\left(ols\right)$. Pick a γ>0, and define the weight vector $w=1/|\hat{\beta }{|}^{\gamma }$. When the variable dimension is higher than the number of samples, w can be obtained by LASSO. By imposing different degrees of penalty to the coefficients of different variables, the LASSO algorithm achieves faster estimation and oracle properties.

Considering that EEG signals are sensitive to noise, we expect a robust weighting of the penalty term, avoiding the bias introduced by the LASSO-based measurement. The symmetric uncertainty, an information entropy-based measurement method, was applied in this work to assess the task relevance of features. We combined the symmetric uncertainty with the spatial information of features to weigh the L1 penalty term in LASSO. The objective function of AS-LASSO is

(5) $$\min_{\beta }{\{∥y-X\beta ∥}_{2}^{2}+\lambda \sum_{i=1}^{p}e^{-\mu s_{i}e_{i}}|\beta_{i}\left|\right\}$$

where $s_{i}$ is the symmetric uncertainty between the *i*-th feature and the class labels, $e_{i}$ is the spatial information of the *i*-th feature, and μ is the adjustment parameter. Different from the LASSO algorithm, we combined the symmetric uncertainty and the spatial information of features to evaluate the weight of the penalty term. This strategy allows the model to consider both the structures between features and the relationship between features and labels, achieving a more robust estimation.

#### 3.2.3. A Learning Algorithm for the Proposed Method

Usually, algorithms, such as the coordinate descent algorithm, the Least Angle Regression algorithm, and the Dual algorithm, are often used to solve the LASSO problem. Considering that the coordinate descent algorithm is faster and less susceptible to noise than other algorithms, we chose to use the coordinate descent algorithm to solve the model. In addition, we transformed solving the AS-LASSO problem into solving the LASSO problem by processing x, following the previous research [40]. The AS-LASSO-based feature selection can be calculated by Algorithm 1.
**Algorithm 1** Algorithm for AS-LASSO-based feature selection.**Input:** X, y, s, λ. **Output:** ${\hat{\beta }}_{j}^{*}$.
  1:Define $x_{j}^{**}=x_{j}/s_{j},j=1,2,\ldots ,p$  2:Solve the LASSO problem for λ,${\hat{\beta }}^{**}=\underset{\beta }{\arg\min}{\{∥y-X\beta ∥}_{2}^{2}+{\lambda ∥\beta ∥}_{1}\}$  3:${\hat{\beta }}_{j}^{*}={\hat{\beta }}_{j}^{**}/s_{j},j=1,2,\ldots ,p$.  4:**return** ${\hat{\beta }}_{j}^{*}$.

When the coordinate descent algorithm is used to update the *j*-th regression coefficient, O(n) calculations are performed at the same time. Therefore, the time complexity of calculating ${\left\{\beta_{j}\right\}}_{j=1}^{p}$ once is O(np). In the experiment, we chose the “LASSO” function provided by the “linear_model” of the “scikit-learn” package to solve the algorithm.

### 3.3. Classification

After obtaining the optimal feature subset, we used traditional classifiers to classify EEG signals. SVM, K-nearest neighbor (KNN), linear discriminant analysis (LDA), random forest (RF) and decision tree (DT) are commonly used classifiers for classification. Considering that the feature subset is characterized by a small sample size, we chose to use SVM as the classifier. It is believed that SVM achieves the best performance in terms of classification performance and computational cost.

## 4. Results and Discussion

In this section, we introduced the EEG datasets we selected and the experimental setup, which showed the implementation details of our presented model, and assessed the performance of the model on the datasets.

### 4.1. EEG Datasets

In this work, we chose three public EEG datasets to assess the performance of our presented model. The details of the datasets are as follows.

**Figure 3:**
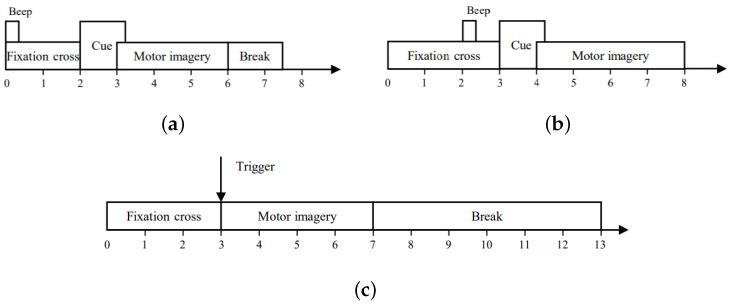
The timing scheme of the datasets. (**a**) The timing scheme of BCI Competition IV Dataset IIa. (**b**) The timing scheme of SMR-BCI Dataset. (**c**) The timing scheme of OpenBMI Dataset.

1. BCI Competition IV Dataset IIa [46]: This dataset contains 22-channel EEG data from nine subjects who were asked to perform four categories of movements (left-hand, right-hand, foot, and tongue). Each subject conducted two sessions on different days. Each session can be subdivided into six runs (48 trials per run). All EEG signals were sampled at 250 Hz and bandpass-filtered between 0.5 and 100 Hz. In this work, only the EEG signals of the left-hand task and the right-hand task were selected for an appropriate comparison. Figure 3a shows the timeline of one trial on this dataset. We limited the time interval of one trial to a period of 2∼6 s.
2. SMR-BCI Dataset [47]: This dataset was provided by the Graz University of Technology in 2014. This dataset was collected from 14 subjects and included EEG signals of the right-hand and foot motor imagery. Each subject recorded 15 channels of EEG signals at a sampling frequency of 512 Hz. Data for each subject included 100 trials without training feedback and 60 trials with test feedback. The timeline of one trial on the SMR-BCI dataset is shown in Figure 3b. In this work, the signal was intercepted through a time window of 4∼8 s.
3. OpenBMI Dataset [48]: This dataset includes 62-channel EEG data from 54 subjects, which are sampled at 1000 Hz. All EEG data are from two sessions conducted on different days. Each session has a training phase and a test phase (100 trials per phase). Each phase contains 50 trials of the right-hand motor imagery task and 50 trials of the left-hand motor imagery task. Figure 3c shows the timeline of one trial on this dataset. In this work, signals are intercepted through a 4-s time window.

### 4.2. Experimental Evaluation

In this work, we chose five-fold cross-validation to evaluate the model’s performance, as adopted by [49]. During the validation process, the original dataset was randomly divided into five subsets to construct the training set and test set for the five experiments. In each experiment, four subsets were selected to construct the training set for training while the remaining subset was used for testing. Meanwhile, we evaluated the model’s performance through metrics such as accuracy, F1-score and precision, which are widely used to evaluate EEG classification models. The definitions of these metrics are as follows:

(6) $$Accuracy=\frac{TP+TN}{TP+TN+FP+FN}$$

(7) $$F1-score=\frac{2TP}{2TP+FP+FN}$$

(8) $$Precision=\frac{TP}{TP+FP}$$

where *TP* (True Positive) represents the number of correctly predicted positive examples, *TN* (True Negative) represents the number of correctly predicted negative examples, *FP* (False Positives) represents the number of falsely predicted positive examples, and *FN* (False Negatives) represents the number of falsely predicted negative examples.

### 4.3. Experimental Setup

In this work, the presented model was implemented using the Python programming language, the “mne” package, and the “scikit-learn” package. EEG signals were processed in a subject-dependent manner and down-sampled to 100 Hz. We decomposed the EEG signals into overlapping multi-scale sub-bands by the fifth-order Butterworth bandpass filters and implemented the spatial domain feature extraction through the “FBCSP” function provided by the “mne” package. Following previous research, we constructed features from spatial filters corresponding to the two largest eigenvalues of each frequency band. Then, we chose the “LASSO” function provided by the “scikit-learn” package to solve the feature selection based on AS-LASSO. The selected optimal subset of features was fed into the SVM classifier provided by the “scikit-learn” package to classify motor imagery tasks. In addition, we evaluated model performance via five-fold cross-validation and reported the average accuracy and average F1-score for each dataset.

### 4.4. Performance of Different Classifiers

We evaluated the performance of FSFF combined with different classifiers. Across all datasets, SVM achieved the highest accuracy score and the highest precision score, as shown in Table 1. In addition, SVM achieved the highest F1-score on the BCI Competition IV Dataset IIa and achieved the second-ranked F1-score on the SMR-BCI Dataset and OpenBMI Dataset. The basic idea of SVM is to solve the hyperplane that can correctly divide the dataset and have the largest geometric separation. SVM provides a small sample learning strategy with a solid theoretical basis.

KNN showed similar scores to SVM on all datasets. On the SMR-BCI Dataset, KNN not only achieved the first F1-score but also had a more stable performance. Unlike other classifiers, KNN does not require complex model training and parameter tuning, which enhances its flexibility and ease of implementation. However, as the dimensions of the data grow, Euclidean distance cannot efficiently measure the similarity in the entire space [50,51]. The performance of KNN is limited by the dimensions of the data. In this experiment, KNN achieved good performance, demonstrating the excellent discriminative feature extraction capabilities of FSFF. On the OpenBMI Dataset, LDA achieved an accuracy that was only lower than SVM and achieved the first F1-score. Compared with SVM and KNN, LDA focuses on all points rather than points that are difficult to classify or nearby points. When there are sufficient samples, LDA shows excellent performance.

### 4.5. Comparisons with State-of-the-Art Models

To assess our presented model, we compared the model with the state-of-the-art models on the three datasets. The comparison models included two machine learning models, four deep learning models, and a hybrid model. A detailed introduction to the comparison models is as follows.

1. FBCSP with SVM [25]: In the model, CSP is used to extract the spatial features of non-overlapping sub-band signals, and the mutual information-based feature selection is used to obtain features matching specific subjects. Finally, the feature subset is fed to the SVM classifier.
2. FBCSP with LDA [52]: The model first divides EEG signals into a series of non-overlapping sub-bands and then applies CSP and LDA classifier to each sub-band, respectively. Finally, score fusion and classification are performed.
3. Deep Convnet [10]: Deep Convnet is expected to achieve an accurate decoding of motor imagery through a general convolutional neural network designed using only a small amount of expert knowledge.
4. EEGnet [11]: EEGNet is a compact convolutional neural network for EEG-based BCI. By building an EEG-specific model using deep and separable convolutions, the model enables the feature extraction and classification of motor imagery.
5. EEG-TCNet [53]: EEG-TCNet is a deep learning-based model for motor imagery. EEG-TCNet achieves excellent performance while requiring a small number of trainable parameters by introducing a temporal convolutional network.
6. MIN2net [49]: MIN2Net is an end-to-end model that integrates deep metric learning into a multi-task autoencoder to learn the compact and discriminative latent representation from EEG.
7. Spectral–Spatial with CNN [54]: Spectral–Spatial with CNN is a motor imagery classification model based on deep convolutional neural networks, whose discriminative features are expressed as a combination of the spectral–spatial input embedding the diversity of the EEG signals.

We first compared the model with FBCSP with SVM and FBCSP with LDA. Typically, EEG signals are set to the 8∼30 Hz range before using the CSP algorithm, covering the frequency range generally considered relevant to motor imagery [55]. Taking into account the optimal frequency sub-bands for specific subjects, the FBCSP algorithm perform non-overlapping band-pass filtering in the 4∼40 Hz range to extract multi-band spatial domain features [25,52]. In this work, we decomposed the signals into overlapping multi-scale sub-bands to further improve the matching of frequency bands to specific subjects. Overlapping multi-scale sub-bands enable the model to better match specific subjects, making the extraction more effective.

In addition, FBCSP with SVM selects features through the mutual information-based feature selection. The mutual information-based feature selection only considers the contribution of a single variable to the model, ignoring the correlation between features. FBCSP with LDA uses a score fusion strategy and does not consider the sparsity of features, resulting in low classification accuracy. We introduced the symmetric uncertainty and the spatial information of features into adaptive LASSO and proposed AS-LASSO for the feature selection algorithm to select the optimal feature subset. The AS-LASSO-based feature selection is a variant of the LASSO-based feature selection, which can simultaneously consider the contribution of multiple features to the model and exhibit superior performance. As shown in Table 2, the classification accuracy of our model amounted to 80.40%, 77.81% and 68.05%, respectively, which is higher than the accuracy of the two machine learning methods.

To further assess the presented model, we compared it with four deep learning models, Deep ConvNet, EEGNet, EEG-TCNet, and MIN2Net. The model based on deep learning is a black box model with the characteristics of autonomously mining discriminative features from data. Although deep learning models avoid reliance on prior knowledge, they require a large amount of data to learn nonlinear features in data and are susceptible to interference from noisy data. Faced with the subject-dependent classification of motor imagery, it is difficult for deep learning models to fully utilize their ability to mine latent features. As shown in Table 2, our presented model achieved a significant lead in accuracy and F1-score compared with Deep ConvNet, EEGNet and MIN2Net. However, EEG-TCNet outperformed our presented model on the BCI Competition IV Dataset IIa, although it was still weaker than our model on the SMR-BCI Dataset and OpenBMI Dataset. The BCI Competition IV Dataset IIa has more training samples, allowing EEG-TCNet to better learn the features hidden in the data. When there are few training samples, our presented model achieves better feature extraction by introducing prior knowledge.

Spectral–Spatial with CNN is a hybrid model that mines the discriminative features through a combination of the Spectral–Spatial input, embedding the diversity of the EEG signals [54]. The performance of Spectral–Spatial with CNN was better than that of deep learning-based models on the SMR-BCI Dataset and OpenBMI Dataset. It is observed that introducing targeted prior knowledge to extract EEG signal features has superior performance compared with feature extraction based on deep learning when there are few samples. Furthermore, the SVM classifier that relies on support vectors for decision making is suitable for small samples. Compared with the SVM classifier, CNN requires a large number of samples for decision making and is prone to interference from any sample. In subject-dependent experiments, the SVM classifier is a better choice when the number of samples is limited.

### 4.6. Ablation Experiments

In this subsection, we performed additional ablation experiments on the BCI Competition IV Dataset IIa, aiming to provide an in-depth analysis of the effectiveness of the presented model. First, we verified the performance of our presented feature extraction strategy. Then, the contribution of the AS-LASSO-based feature selection was verified by comparing it with the LASSO-based feature selection and the adaptive LASSO-based feature selection.

#### 4.6.1. Effect of the Feature Extraction Strategies

To illustrate the effectiveness of our feature extraction strategy, we removed the strategy from our presented model, which only adopted a single sub-band to extract CSP-based features (i.e., CSP). We evaluated the performance of CSP in the range of 4∼40 Hz, demonstrating the role of decomposing EEG signals into sub-bands. As shown in Table 3, our strategy achieved much higher scores than the feature extraction based on the single sub-band in both accuracy and F1-score. The experimental results show that decomposing the EEG signal into multiple sub-bands helps extract the discriminative features.

Furthermore, we compared the proposed strategy with the strategy decomposed into non-overlapping sub-bands (i.e., FBCSP). Generally, FBCSP decomposes the EEG signals into non-overlapping sub-band signals in the 4∼40 Hz range and a bandwidth of 4 Hz to match specific subjects. Compared with FBCSP, we set up multi-scale frequency bands and made the frequency ranges overlap. As shown in Table 3, our strategy achieved significant improvements compared to FBCSP in accuracy, F1-score and precision with scores of 80.40%, 79.10% and 81.52%. The experimental results demonstrate that overlapping multi-scale sub-bands enable the model to better match the brain activities of different subjects.

#### 4.6.2. Effect of the Feature Selection Methods

**Figure 4:**
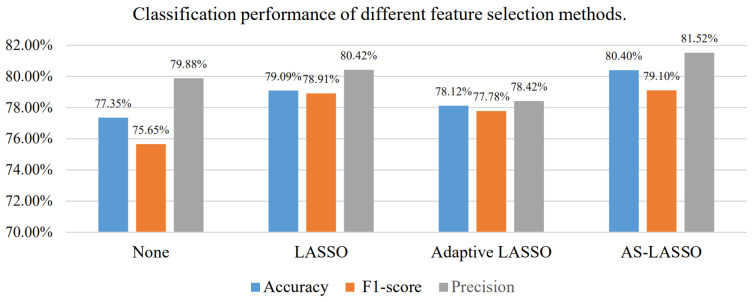
Classification performance of different feature selection methods.

We compared the proposed AS-LASSO-based feature selection with the other two LASSO-based feature selections, as shown in Figure 4. Compared with no feature selection, all three feature selection methods achieved higher accuracy and F1-scores. Among them, the AS-LASSO-based feature selection achieved the best performance with an accuracy increase of 1.31% compared to the LASSO-based feature selection.

The adaptive LASSO-based feature selection was lower than the LASSO-based method in accuracy, F1-score and precision. Furthermore, the precision score of the adaptive LASSO-based feature selection was lower than that without feature selection. The adaptive LASSO-based feature selection increased the probability that the model misjudges the sample as a positive class. The introduction of adaptive weights accelerates the convergence of the LASSO algorithm and further aggravates the problem of selecting any one of the similar features. We introduced the symmetric uncertainty and the spatial information of features to optimize the weight measurement of the penalty term in adaptive LASSO and achieved superior performance.

We further compared three measurement methods of penalty term weight: the symmetric uncertainty, the spatial information of features, and LASSO, as shown in Table 4. The symmetric uncertainty measures the task relevance of features, thereby assessing their importance. Compared with the LASSO-based method, the symmetric uncertainty has a more robust measurement performance and enables adaptive LASSO to achieve better effects.

The spatial information of the features was provided by the eigenvalues when constructing the spatial filter. The value of the eigenvalues is related to the significance of the feature and the channel corresponding to the feature. By calculating the eigenvalues of extracted features in different frequency bands, spatial information evaluates the importance of features from the significance of brain activity in different regions. We used spatial information to evaluate the weight of the penalty term and demonstrated its good performance. To further improve the performance of feature selection, we fused the symmetric uncertainty with the spatial information and proposed AS-LASSO. As shown in Table 4, the experimental results show that the strategy of integrating the symmetric uncertainty and the spatial information can effectively improve the performance of feature selection.

### 4.7. Visualization of Selected Features

**Figure 5:**
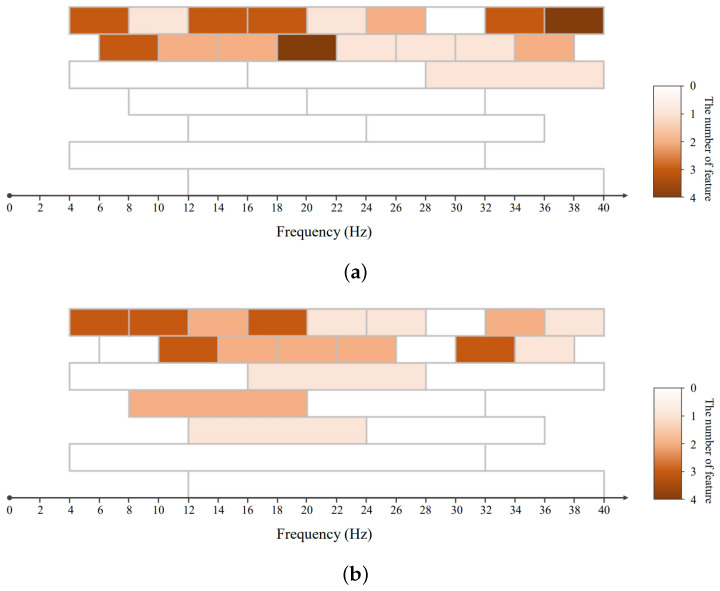
The visualization of the features selected by the AS-LASSO-based feature selection. (**a**) The example from subject 1. (**b**) The example from subject 3.

Currently, a large amount of research has focused on improving the quality of spatial features of EEG signals, such as FBCSP. However, many studies perform feature extraction based on fixed frequency bands. These methods ignore the individual differences in the frequency ranges of brain activity, making it difficult to flexibly cover the actual brain activity during the execution of motor imagery tasks. To improve the quality of spatial domain features, we decomposed the EEG signal into overlapping multi-scale sub-bands and learned features matching specific subjects through the AS-LASSO-based feature selection. The decomposition strategy is shown in Figure 2. We randomly selected Subject 1 and Subject 3 from the BCI Competition IV Dataset IIa and visualized the features selected by the AS-LASSO-based feature selection on the two subjects. The visualization of the features is shown in Figure 5.

Through the visualization, it can be observed that there are differences in the sub-bands of features selected by the AS-LASSO-based feature selection for different subjects. The selected features on both subjects are mainly distributed in the sub-bands of 4 Hz bandwidth, providing detailed information on the EEG signals. A small number of features are distributed in the sub-band of 8 Hz bandwidth, providing complementary information. In addition, we extracted the spatial features of sub-band signals in the frequency range of 4∼32 Hz and the frequency range of 12∼40 Hz. The sub-band of 4∼32 Hz is similar to the frequency bands used in most CSP algorithms. However, the model did not select the features extracted from the sub-band of 4∼32 Hz and 12∼40 Hz for some subjects. Features extracted in the narrow frequency bands have better discriminability. The experimental results show that decomposing EEG signals into sub-bands of different frequency scales can better mine motor imagery-related information of brain activity.

## 5. Conclusions

In this work, we presented a flexible motor imagery classification model based on FSFF to improve the model’s matching to specific subjects. FSFF set up a set of overlapping bandpass filters to obtain sub-band signals of different frequency scales and effectively fused the multi-scale spatial features through the AS-LASSO-based feature selection. The AS-LASSO-based feature selection introduced the symmetric uncertainty and the spatial information to mine the discriminative features, making full use of the task relevance and structural information of features. We applied FSFF to three datasets (BCI Competition IV Dataset IIa, SMR-BCI dataset, and OpenBMI dataset). The experimental results demonstrated that our presented model outperformed the state-of-the-art methods in motor imagery classification.

Although FSFF shows excellent performance in feature engineering, we only considered individual differences in the frequency domain and spatial domain. In subsequent studies, we expect to segment the EEG signals by increasing time windows to match the responses of different subjects.

## Figures and Tables

**Table 1:** Classification performance (in %, ± standard deviation) of different classifiers.

Dataset	Classifier	Accuracy	F1-Score	Precision
BCI Competition IV Dataset IIa	KNN	79.03 ± 13.69	78.77 ± 15.28	80.28 ± 15.06
	LDA	73.77 ± 13.12	70.88 ± 19.93	77.73 ± 15.08
	RF	71.81 ± 11.87	68.46 ± 15.23	77.42 ± 15.63
	DT	67.15 ± 11.85	68.18 ± 11.47	67.79 ± 13.45
	SVM	**80.40 ± 13.42**	**79.10 ± 18.55**	**81.52 ± 15.31**
SMR-BCI Dataset	KNN	77.79 ± 15.35	**76.52 ± 17.23**	79.90 ± 15.81
	LDA	72.31 ± 16.53	71.66 ± 18.09	73.79 ± 17.48
	RF	73.71 ± 17.02	70.09 ± 21.20	77.51 ± 18.18
	DT	71.95 ± 17.89	72.35 ± 17.73	73.14 ± 19.18
	SVM	**77.81 ± 15.08**	74.60 ± 19.01	**82.78± 15.73**
OpenBMI Dataset	KNN	67.12± 16.32	66.79 ± 16.95	67.59 ± 16.47
	LDA	67.39 ± 15.84	**68.81 ± 30.95**	67.95 ± 16.10
	RF	65.95 ± 15.59	62.88 ± 17.74	68.20 ± 16.47
	DT	63.63 ± 14.68	63.55 ± 14.97	64.17 ± 15.15
	SVM	**68.05 ± 16.54**	67.91 ± 17.75	**68.43 ± 16.87**

The best results are highlighted in bold.

**Table 2:** Comparisons (in %, ± standard deviation) with state-of-the-art models.

Dataset	Method	Accuracy	F1-Score
BCI Competition IV Dataset IIa	FBCSP with SVM	75.93 ± 14.76	74.49 ± 18.47
	FBCSP with LDA	73.75 ± 18.22	75.72 ± 25.59
	Deep ConvNet	64.34 ± 17.89	60.17 ± 22.70
	EEGNet	65.68 ± 18.22	64.18 ± 25.59
	EEG-TCNet	**84.15 ± 14.01**	**84.49 ± 13.54**
	MIN2Net	65.46 ± 15.60	64.54 ± 18.35
	Spectral-Spatial with CNN	76.84 ± 13.63	76.95 ± 15.28
	Ours	80.40 ± 13.42	79.10 ± 18.55
SMR-BCI Dataset	FBCSP with SVM	74.26 ± 17.45	70.80 ± 22.26
	FBCSP with LDA	74.38 ± 19.48	71.87 ± 21.95
	Deep ConvNet	61.52 ± 15.87	55.90 ± 21.48
	EEGNet	67.76 ± 17.96	68.05 ± 20.96
	EEG-TCNet	68.50 ± 20.13	67.67 ± 21.62
	MIN2Net	64.88 ± 15.09	62.70 ± 16.56
	Spectral-Spatial with CNN	75.88 ± 17.01	69.80 ± 26.99
	Ours	**77.81 ± 15.08**	**74.60 ± 19.01**
OpenBMI Dataset	FBCSP with SVM	66.69 ± 16.22	65.88 ± 18.41
	FBCSP with LDA	66.05 ± 16.21	65.73 ± 17.56
	Deep ConvNet	60.17 ± 16.52	61.69 ± 18.38
	EEGNet	60.42 ± 17.08	56.81 ± 23.49
	EEG-TCNet	63.32 ± 16.36	62.73 ± 17.94
	MIN2Net	59.78 ± 13.92	62.17 ± 14.22
	Spectral-Spatial with CNN	65.33 ± 15.98	67.56 ± 15.81
	Ours	**68.05 ± 16.54**	**67.91 ± 17.75**

The best results are highlighted in bold.

**Table 3:** Classification performance (in %) of different feature extraction strategies.

Feature Extraction	Accuracy	F1-Score	Precision
CSP	69.09	64.41	72.44
FBCSP	78.10	77.89	79.54
Our strategy	**80.40**	**79.10**	**81.52**

The best results are highlighted in bold.

**Table 4:** Classification performance (in %) of different weight measurement methods.

Weight Measurement	Accuracy	F1-Score	Precision
LASSO	78.12	77.78	78.42
Symmetric uncertainty	79.72	78.88	80.76
Spatial information	79.18	77.39	**81.92**
Our strategy	**80.40**	**79.10**	81.52

The best results are highlighted in bold.

## Data Availability

Publicly available datasets were analyzed in this study. The BCI Competition IV Dataset IIa can be foundhere: https://www.bbci.de/competition/iv/ (accessed on 5 November 2023). The SMR-BCI Dataset can be found here: http://bnci-horizon-2020.eu/database/data-sets (accessed on 5 November 2023). The OpenBMI Dataset can be found here: http://gigadb.org/dataset/view/id/100542/File_page (accessed on 5 November 2023).
